# Molecular Mechanisms of Dementia

**DOI:** 10.3390/ijms241713027

**Published:** 2023-08-22

**Authors:** Mariagiovanna Cantone

**Affiliations:** Neurology Unit, Policlinico University Hospital “G. Rodolico-San Marco”, 95123 Catania, Italy; m.cantone@policlinico.unict.it

The various forms of dementia and the other neurodegenerative disorders that affect memory, cognition, and behavior have become a public health priority across the developed world. It has been estimated that the number of people with dementia will double every 20 years. Currently, one of the most pressing clinical challenges is to discriminate the different forms of dementia, especially during their earliest stages, from normal brain aging. The presence of clinical similarities between different forms of dementia and other neurodegenerative disorders raises the question as to whether common molecular pathways might explain shared clinical symptoms. Although several mechanisms have been hypothesized, at both the cellular and neuronal network levels [1,2], the exact molecular mechanisms that lead to dementia and other neurodegenerative disorders are not well-understood.

This Special Issue of the International Journal of Molecular Sciences collects both original research articles and review papers that deal with the emerging molecular mechanisms of dementia. It is known that neurodegenerative diseases are usually related to protein deposits or misfoldings, eventually leading to chemical changes and progressive neuronal loss in the brain and spinal cord, thus inducing loss of function. The most common types of proteins that are implicated in the formation of these aggregates are the amyloid-β, tau protein, α-synuclein, and prion protein.

Alzheimer’s disease (AD) is the cause of almost all cases of dementia with symptoms dominated by memory loss, impaired behavior, and judgmental disorders [3]. Extracellular neuritic plaques and intercellular neurofibrillary tangles (NFTs), composed of aggregated β-amyloid (Aβ) and hyperphosphorylated tau protein, are the well-known neuropathological hallmarks of AD. Moreover, recently, increasing evidence suggests that in AD and other tau-related pathologies, the pattern of tau deposition follows a stereotypical progression between anatomically connected brain regions [4], in a “prion-like” manner, and that the seeding and spreading of pathological tau drive progressive neurodegeneration.

Isidro Ferrer and colleagues [5] identified tau pathology seeded through the intra-hippocampal inoculation of sarkosyl-insoluble fractions from AD, Pick’s disease (PiD), and globular glial tauopathy (GGT) in the hTau transgenic mice on tau deposits. These mice expressed the six human tau isoforms, with a high predominance of 3Rtau over 4Rtau. Authors found that tau strains from various tauopathies produced different patterns of AT8 immunostaining and 3R-tau and 4R-tau deposits induced by tau seeds from 4R and 3R tauopathies, which also depend on the host tau.

Similarly, an intriguing attempt to characterize the molecular mechanisms possibly linked to other forms of neurodegenerative diseases can be found in the research of Cheng and colleagues [6]. At present, there is no cellular model that perfectly demonstrates the pathological changes seen in amyotrophic lateral sclerosis (ALS) or frontotemporal dementia (FTD). The authors revealed two different common cellular models of ALS and FTD. The first was an oxidative stress model using HEK-293 cells overexpressing the DNA-binding protein 43 (TDP-43) wild type treated with a chronic dose of sodium arsenite. The second used SH-SY5Y neuroblastoma cells, stably expressing a disease-relevant mutant form of TDP-43 (TDP-43 M337V). They used Western blotting to assess whether TDP-43 and stress granule and ubiquitin localization and solubility in these models mirrored that seen in human ALS and FTD. The sodium arsenite model recapitulated most aspects of TDP-43, stress granule, and ubiquitin pathology, whereas the mutant model only displayed some aspects. The data support the notion that different tau strains produce different patterns of neuronal tau deposition and seeding, as well as that tau seeding depends on the host tau. 

Cellular models enable the precise and detailed study of the putative mechanisms responsible for the progression of tauopathies. Understanding such mechanisms is crucial for shedding light on the etiology of human tauopathies and also for identifying novel molecular targets for innovative therapies. In this context, Julia Sala-Jarque and colleagues [7] describe the development of cellular models of tau pathology and the “prion-like” nature of pathological tau. In particular, the authors demonstrate advantages and disadvantages of in vivo rodent models, 2D mammalian immortalized cell lines, microfluidic devices, such as murine neural cells, 2D iPSC-derived neurons, and 3D cerebral organoids.

The comprehensive review by Loh and Reiter [8] for the first time adequately summarizes the methodology for studying the role of melatonin as an integral regulator of biological rhythms and analyzes in depth the role of this hormone in providing synergistic relationships in nature between two major environmental factors (i.e., water and light). Light, water, and melatonin constitute an ancient synergy that ensures adequate protein hydration to prevent aberrant phase separation.

Finally, the narrative review by Janusz Wiesław Błaszczyk [9] aimed to explain the etiology of dementia and AD from the perspective of energy and cognitive metabolism dysfunction in the aging brain. Starting from the evidence that the brain is a highly energy-demanding structure whose functioning depends primarily on a stable and efficient energy supply, the author clearly describes how energy metabolism is essential for the networking of limbic areas implicated in cognitive processes.

Despite a plethora of studies, only a few drugs are currently available for AD. First of all, in accordance with evidence of a cholinergic deficit in AD responsible for cognitive impairment, the most commonly prescribed treatments for AD are acetylcholinesterase inhibitors, such as donepezil or galantamine. Beyond its impact on cognitive function, this cholinergic deficit is linked to the behavioral and psychological symptoms of dementia (BPSD). Mahmoudi and colleagues [10] reviewed the established relations between exposure to anticholinergic drugs and the onset or persistence of BPSD, thus highlighting the pathophysiology and implications for therapeutic management.

The paper by Wojtunik-Kulesza and colleagues [11] presented the amyloid hypothesis as a cornerstone of therapy, as well as the latest information about aducanumab. The activity of this drug is targeted at the amyloid, which is considered one of the main causes of AD. The FDA’s acceptance of this drug caused much controversy in the scientific community. Several studies revealed the activity of the drug against β-amyloid, although the drug has more negative than positive features.

The manuscript by Sekikawa and colleagues [12] reviews the current knowledge of properties and mechanisms of action of S-equol for improving memory and cognitive function. The first part of the paper describes soy isoflavones and the formation of S-equol as well as the mechanism of action and its bioavailability. The main part of the manuscript is related to the cognitive benefits of S-equal, based on both preclinical and clinical studies. The authors provided an overview contaning several aspects of S-equol, connecting it to various physiological and brain-related functions and also to a neuroprotective role in health and disease, by examining its involvement in antioxidant to neurodegenerative diseases, arterial stiffness, and white matter defects, as well as the involvement of the estrogen receptor.

Repetitive transcranial magnetic stimulation (rTMS) is a non-invasive neuromodulation technique that is also applied in cases of mild cognitive impairment (MCI) and AD, among other cognitive and neuropsychiatric disorders [13,14,15,16]. However, the neurobiological mechanisms underlying the rTMS therapeutic effects are still only partially investigated. The double-blind, sham-controlled study by Cirillo and colleagues [17] provided preliminary data about the effects of rTMS on cognitive functions and the change in blood levels of plasmatic matrix metalloproteinases, which are enzymes linked to apoptosis, among other functions, in MCI patients. The authors found that rTMS changed the levels of plasmatic matrix metalloproteinases and improved visuo-spatial abilities in MCI participants.

Parkinson’s disease (PD) is a progressive neurodegenerative condition, with rigidity, bradykinesia, postural instability, gait dysfunction, and tremors being the main clinical features. Non-motor features, such as dementia, hyposmia, and gastrointestinal abnormalities, are often present in the course of the disease. The progressive degeneration of the nigrostriatal dopaminergic pathway leads to significant neuron loss in the substantia nigra pars compacta neurons and the depletion of dopamine with the a-synuclein aggregates, known as Lewy bodies or neurites, in several areas of the central nervous system. In the review by Deyell and colleagues [18], the authors combined and summarized published data about the role of α-synuclein in its interaction with the microglia. In particular, they focused on the chronological cascade of microglial events in synucleinopathies, describing the potential downstream effects of the α-synuclein interaction with microglia.

Lastly, Martínez-Pinteño A and colleagues [19] analyzed the protein levels of nitric oxide synthase (NOS) isoforms in the adult prefrontal cortex (PFC) and the ventral hippocampus (HPC) of a postnatal ketamine-induced murine model of schizophrenia. In the model mice received clozapine (CLZ) or the novel metabotropic glutamate receptor modulator JNJ-46356479 (JNJ) during adolescence. Endothelial NOS and neuronal NOS increased under ketamine administration in PFC and in HPC and decreased in CLZ or JNJ treatments. Conversely, inducible NOS increased under JNJ treatment with respect to ketamine induction in the HPC and PFC, suggesting a misbalance of the NOS system following NMDAr antagonist administration.

In conclusion, the studies will provide further diagnostic and therapeutic frames for the characterization of patients with dementia and neurodegenerative diseases. Future studies are needed to validate new hypothesis and to expand the present findings to other neurodegenerative diseases or neuropsychiatric disorders with cognitive involvement.
